# Liver failure after treatment with inotuzumab and polychemotherapy including PEG-asparaginase in a patient with relapsed Philadelphia chromosome–negative acute lymphoblastic leukemia

**DOI:** 10.1007/s00277-023-05495-w

**Published:** 2023-11-24

**Authors:** Daniel Fischer, Rosa Toenges, Kati Kiil, Sabine Michalik, Axel Thalhammer, Gesine Bug, Nicola Gökbuget, Fabian Lang

**Affiliations:** 1 Department of Medicine, Hematology and Oncology, University Hospital, Goethe University Frankfurt, Frankfurt am Main, Germany; 2 Senckenberg Institute of Pathology, University Hospital, Goethe University Frankfurt, Frankfurt am Main, Germany; 3 Department of Radiology, University Hospital, Goethe University Frankfurt, Frankfurt am Main, Germany

**Keywords:** Acute lymphoblastic leukemia, Extramedullary relapse, Inotuzumab ozogamicin, Pegaspargase, Liver failure

## Abstract

We present the case of a 58-year-old female patient who presented with an extramedullary B-ALL relapse after prior allogenic HSCT and blinatumomab therapy. The patient died from complications of a drug-induced acute liver failure after a salvage therapy combining inotuzumab ozogamicin (InO)-based induction followed by consolidation with high dose MTX and pegaspargase based on the GMALL protocol for older ALL patients. After a diagnosis of the extramedullary relapse in the form of a retro vesical chloroma, the patient received an individualized multi-agent chemotherapy based on induction chemotherapy for older patients in combination with InO. After four administrations of InO, in combination with vincristine, dexamethasone, cytarabine, and cyclophosphamide, CT-imaging showed a reduction in volume of the chloroma and response to therapy. Consolidation with high-dose methotrexate and pegaspargase was administered. The patient developed toxic liver damage manifested by hyperbilirubinemia and progressive hepatic encephalopathy. The diagnostic criteria for VOD were met, and therapy with defibrotide was initiated. Liver biopsy revealed no histological signs of VOD but instead steatohepatitis indicative of drug-induced toxicity. The patient ultimately died of hemorrhagic shock through postinterventional hemorrhage after liver biopsy. In conclusion, although InO shows promising results in the therapy of r/r ALL with and without additional chemotherapy, the combination with MTX and pegaspargase in an intensively pretreated patient with relapse after HCST may impart an increased risk for liver-related toxicity. Special caution is required when assessing fitness for further liver toxic regimens. A key takeaway is also the reminder that InO can cause liver damage not only in the form of VOD but also through direct hepatocellular toxicity.

## Background

Toxic liver injuries caused by chemotherapeutic agents, including veno-occlusive disease (VOD), are rare but potentially serious and life-threatening complications. Their prevention requires a high degree of experience and clinical vigilance. VOD, also referred to as hepatic sinusoidal obstruction syndrome (SOS), mainly occurs after allogenic hematopoietic stem cell transplantation (allogenic HSCT) [1, 2]. It is a form of systemic endothelial disease, causing refractory thrombocytopenia, jaundice, hepatomegaly, and ascites. There are some differences between VOD in adults and in children. In this case report, we will only refer to the adult form. The severeness of VOD can vary from relatively mild to cases which progress rapidly and cause multiorgan failure and death. The established risk factors for VOD may be divided into pretransplant patient characteristics (e.g., prior liver disease, lung disease, and age) and transplant-related factors (e.g., graft source, GvHD prophylaxis, conditioning regime). The main cause of VOD is seen in intensive myeloablative conditioning, especially with alkylating agents [3]. Since the use of monoclonal antibodies conjugated with calicheamicin (e.g., gemtuzumab ozogamicin and inotuzumab ozogamicin), there has been accumulating evidence of increased risk for VOD, especially for patients who undergo HSCT subsequently [4]. So far, there is only one empirically proven therapy for VOD, defibrotide [5]. However, this therapy is associated with a significantly increased risk of bleeding, which is why the indication requires scrutiny. A central problem is the differentiation between VOD and its differential diagnoses such as GvHD, drug-induced liver injury (DILI), viral or fungal infections, and blast infiltration. Since VOD is mainly diagnosed on the basis of non-specific clinical criteria, comprised in the EBMT diagnostic criteria for adults [6], clinicians have to deal with diagnostic uncertainty. In this case report, we cover how the fate of a patient was decided by the search for the right diagnosis and treatment strategy.

## Case report

**Fig. 1:**
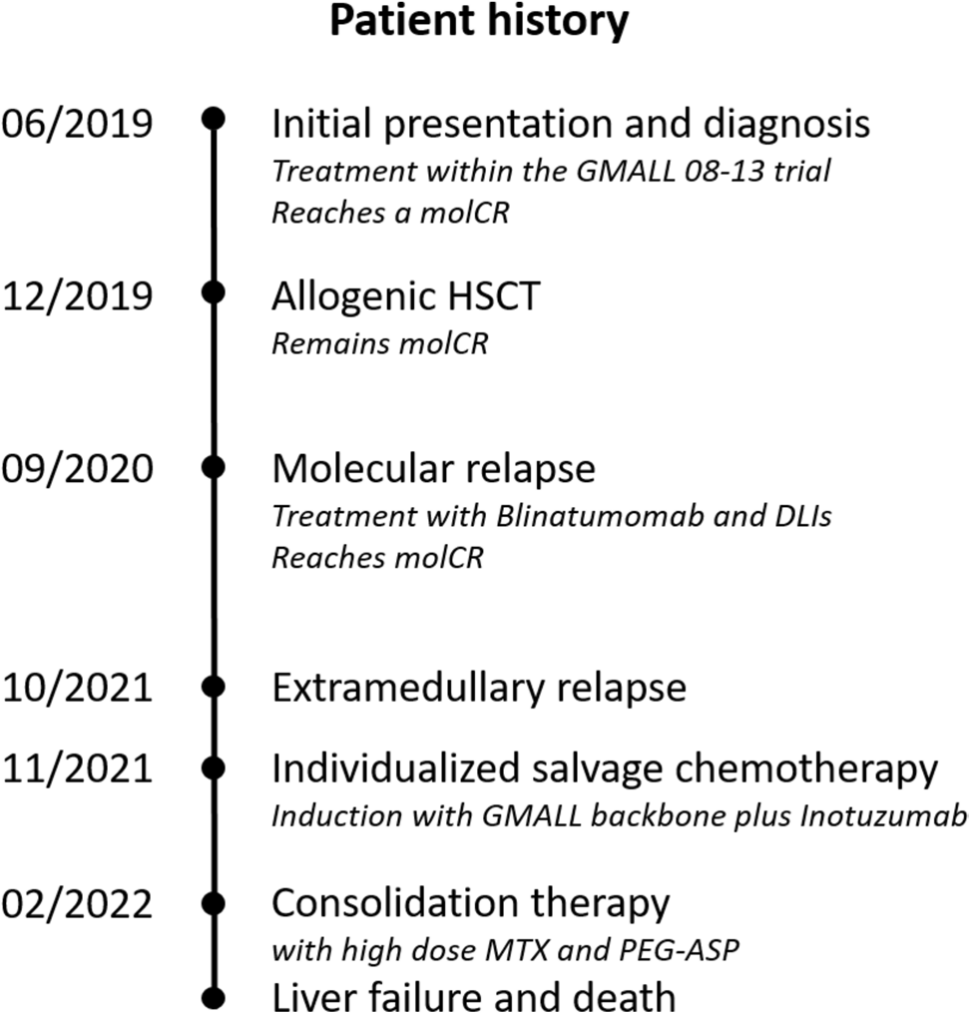
Timeline of the patients’ history

In mid-2019 a 55-year-old woman presented to the clinic as an outpatient. She reported knee pain, severe fatigue, and night sweats. Her initial blood count showed the following: leukocytes 67/nL, platelets 251/nL, and a Hb of 10 g/dL. Furthermore, 60% of the peripheral cells were lymphoblasts. Bone marrow aspiration found a blast percentage of 49%. Cytogenetic examination revealed cell lines with normal karyotype, cells with trisomy 22, and homozygous CDKN2A deletion, del(9)(p21p21). Immunological diagnostics revealed 4.9% CD20 expression. An aberrant expression of CD33 was found. Molecular genetic testing showed no evidence of BCR-ABL, MLL-AF4, and MLL-ENL. A cerebrospinal fluid (CSF) puncture revealed no evidence of CNS infiltration. There were no signs of extramedullary involvement. In the synopsis of the findings, the patient was diagnosed with a high-risk Ph neg. pro-B acute lymphoblastic leukemia (ALL). An overview of the further development can be found in Fig. 1.

In June 2019, the patient was enrolled in the GMALL 08–13 trial for first-line treatment [7]. She received pre-phase, as well as induction 1 and 2. Chemotherapeutic treatment included the administration of, among other, cyclophosphamide, rituximab, and pegaspargase (2000 U/m^2^). In addition, the patient received prophylactic intrathecal MTX. During induction 2, the second administration of pegaspargase was omitted, as there was a significant increase in bilirubin (CTCAE grade 3) after the first administration. On day 22, a bone marrow puncture showed a blast percentage of 5%. After the second induction, the patient achieved a hematological complete remission (CR) with sustained minimal residual disease (MRD) positivity at a level of < 3 × 10^−5^ (below the quantitative range). After subsequent consolidation 1, a molecular CR (MRD negative with a sensitivity of 10^−4^) was achieved. As bridging-to-transplant, a dose-reduced consolidation 2 was given.

An allogenic peripheral blood stem cell transplant (PBSCT) from an HLA-identical unrelated donor followed in December 2019 after conditioning with fludarabine and 8 Gy total body irradiation (TBI). The patient showed a complete chimerism in peripheral blood on day + 14. The transplantation was performed without major complications, although a skin GVHD stage 1 developed. The patient was discharged after successful therapy. Subsequent MRD testing was negative.

Nine months after HSCT, a routine bone marrow examination showed MRD positivity at a level of 5 × 10^−3^. The molecular relapse was treated with blinatumomab. During the administration, the patient showed neurological abnormalities, including altered gait and writing. Neurological side effects are a known complication of blinatumomab. Symptom control was achieved by dose reduction. The relapse was successfully treated, and the patient regained a molecular CR. In addition, 2 donor lymphocyte infusions (DLI) were administered prophylactically. The first with 0.5 and the second with 1 × 10^6^ cells/kg bodyweight. No signs of GVHD developed.

After a year without further therapy and without evidence of MRD in the bone marrow, the patient developed clinical symptoms. A urinary stasis was caused by a retrovesical chloroma. The extramedullary relapse was confirmed via biopsy showing malignant B-cells which were negative for CD20. Bone marrow examination showed MRD at a level of 1 × 10^−4^. The patient first required urological treatment. After the insertion of two double J-catheters, she was readmitted for hematological treatment. It was decided to treat her with a salvage therapy combining InO-based induction followed by consolidation with high-dose MTX and pegaspargase based on the GMALL protocol for older ALL patients [8]. The available evidence suggests a good efficacy of InO in patients with relapsed/refractory ALL and limited data indicate efficacy in extramedullary disease. [9, 10]

**Fig. 2:**
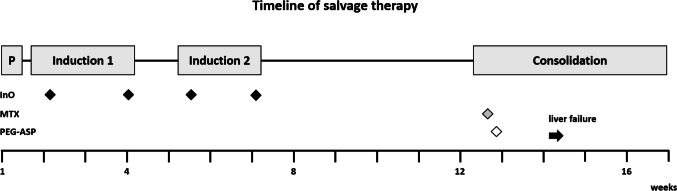
Schematic representation of the therapy sequence. Pre-phase (P) including cyclophosphamide and intrathecal MTX. Induction 1: Including rituximab, vincristine, dexamethasone, InO, and intrathecal triple therapy (MTX, cytarabine, and dexamethasone). Induction 2: Including rituximab, cyclophosphamide, cytarabine, InO, and intrathecal triple therapy. Consolidation: Including rituximab, high-dose MTX (1000 mg/m^2^) and pegaspargase (1000 IE/m^2^)

The relapse therapy started in November 2021 (Fig. 2). The patient received a pre-phase, induction 1, and induction 2 successively in an inpatient and outpatient setting. During the administration of cyclophosphamide during the pre-phase, there was a brief increase in transaminases with GOT at 215 U/L and GPT at 129 U/L. However, the transaminases quickly normalized and there was only a slight increase in bilirubin. The induction 1 comprised vincristine, dexamethasone, and 2 doses of InO; the first dose with an amount of 0.6 mg/m^2^ and a second with 0.3 mg/m^2^. As recommended for therapy with InO, the patient received prophylaxis with ursodiol for the entire period of therapy [11]. Overall, the therapy was well tolerated by the patient, and no complications occurred. The patient was discharged and received the second half of induction 1 in an outpatient setting. In December, induction 2 of the protocol was followed with cyclophosphamide, AraC, and two additional doses of InO, each at 0.3 mg/m^2^. No major complications occurred. A CT scan of the abdomen was obtained after the therapy. Imaging revealed a marked reduction in the volume of the chloroma.

**Fig. 3:**
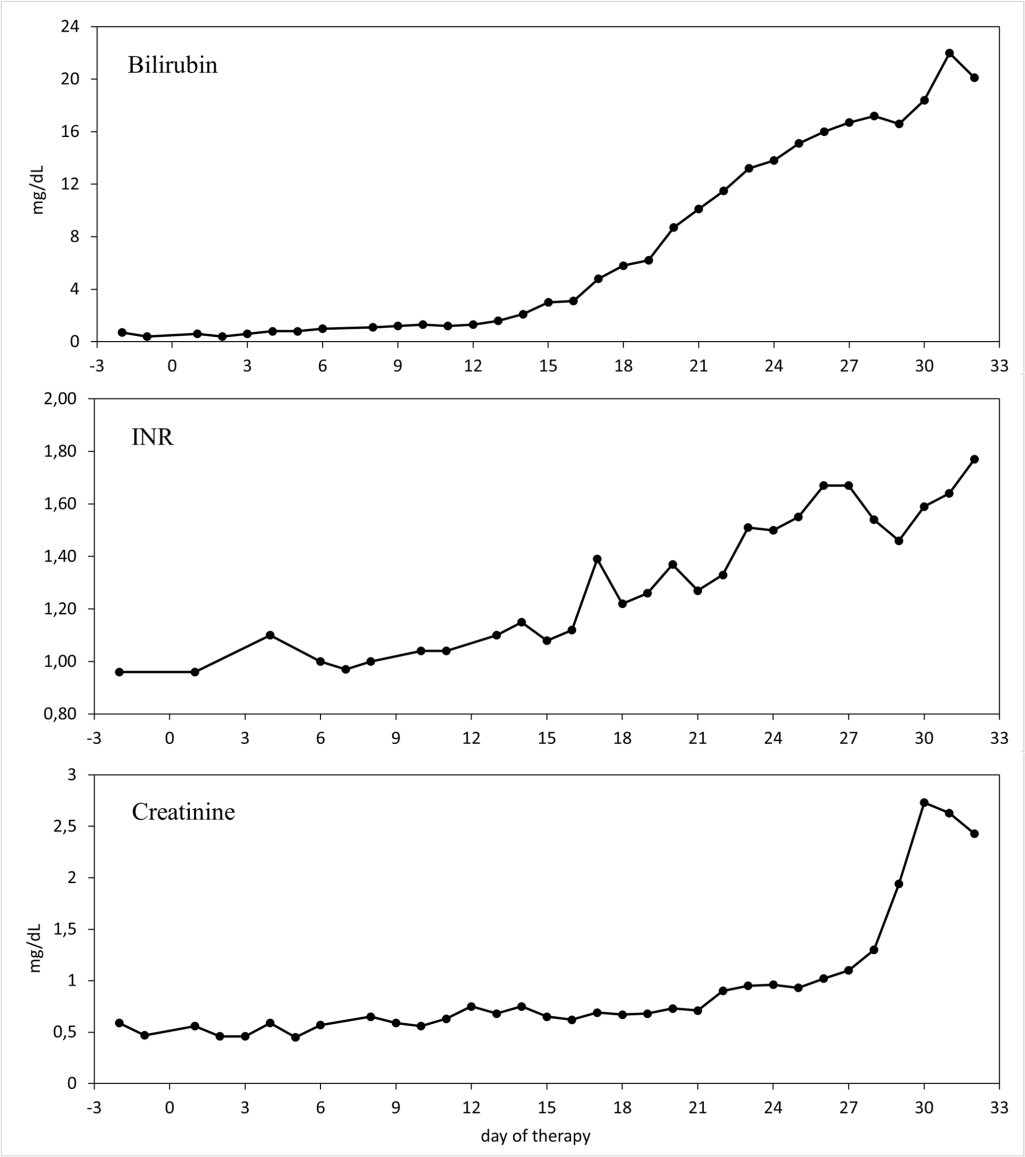
Blood tests during the last chemo protocol with high dose MTX and pegaspargase. A simultaneous increase in bilirubin, INR, and creatinine is the result of progressive liver failure

**Fig. 4:**
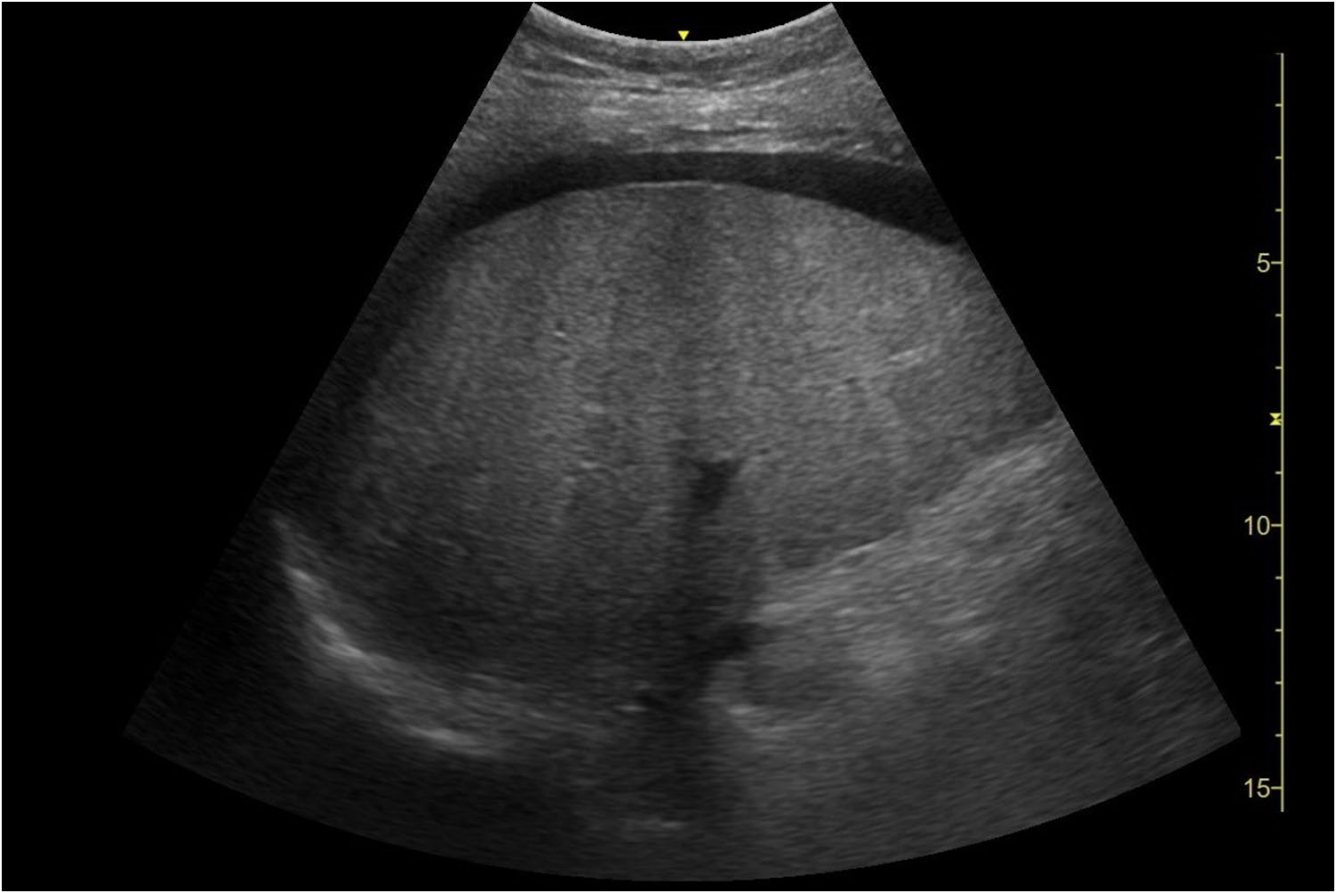
A sonographic image of the liver prior to biopsy. Minimal perihepatic ascites is present

**Fig. 5:**
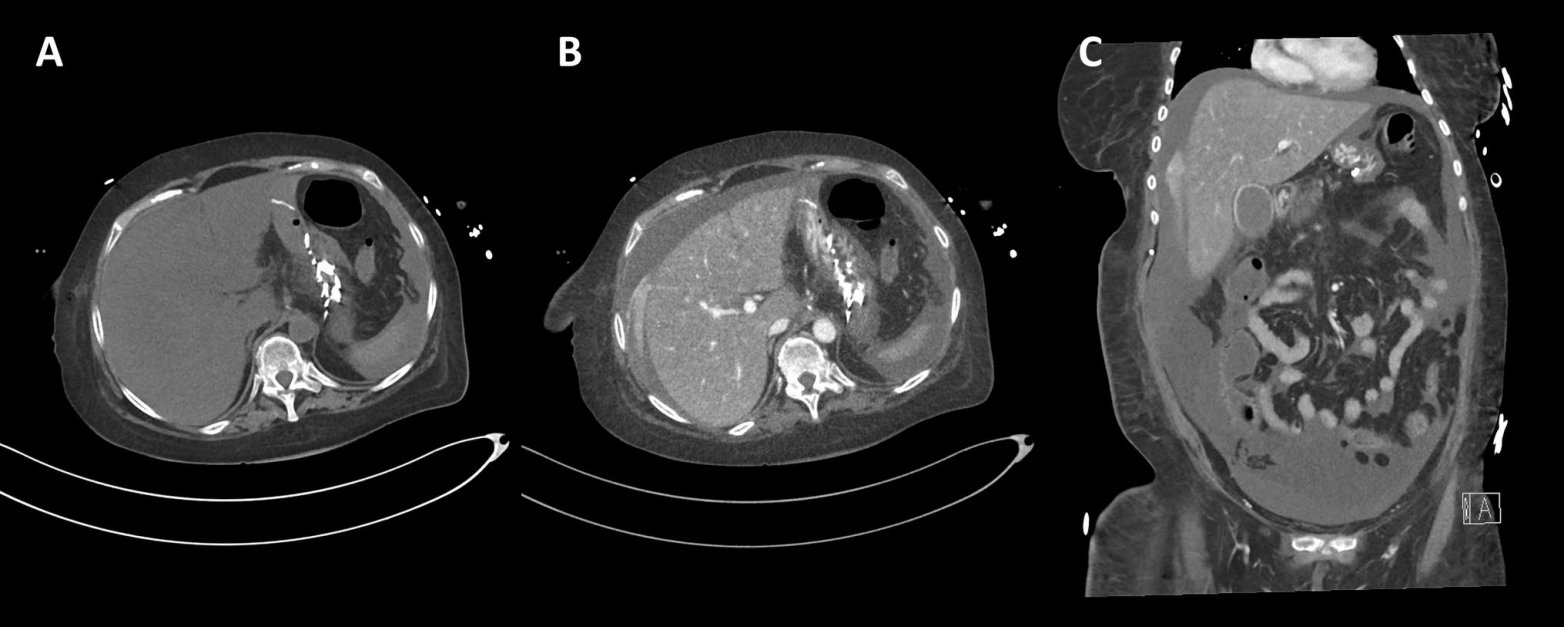
CT angiography of the abdomen (**A** native, axial plane, slice thickness 5 mm; **B** venous phase, axial, 1.5 mm; **C** venous phase, coronal, 3 mm). Hemorrhage originating from the liver capsule near segment six creates a contrast in the ascites. A small subcutaneous air pocket indicates the puncture site

At the end of January 2022, the patient was admitted for consolidation 1, a cycle with high-dose MTX and pegaspargase. Starting with rituximab at day 0, high-dose MTX (1000 mg/m^2^) as 24-h infusion and pegaspargase (1000 IE/m^2^) were administered on days 1 and 2. MTX clearance was normal. On day 4 of therapy, the patient showed a reduced general condition; in particular, an onset of oral mucositis was noticeable. The pain was well controlled with oral oxycodone, but the mucositis worsened up to grade 4. With chronically low platelets and reduced antithrombin III (AT III) activity, the patient was symptomatic with recurrent epistaxis. Regular platelet concentrate infusions and AT III substitution were given. On day 10, bilirubin increased for the first time and the patient developed severe pruritus, tempting her to scratch her skin open. Neuropathy with tingling paresthesia in the fingers also developed. On day 12, hepatotoxicity manifested itself for the first time by morning hypoglycemia. Bilirubin had risen to 1.3 mg/dL. On day 15, a sonography of the abdomen was performed showing free fluid perihepatic, perisplenic, and the lower abdomen. The liver parenchyma, bile ducts, and hepatic vessels were found to be normal. Diagnostic PCRs for hepatotropic viruses (CMV, EBV, HHV6 and 8, HAV, HBV, HCV, and HEV) were negative. The patient continued to develop a pronounced pressure pain in the right upper abdomen even with superficial palpation, so all criteria of VOD were now met. On the same day, a therapy with defibrotide (4 × 400 mg i.v. over 2 h) was started. In the following days, the patient retained more and more fluid. Her abdominal girth increased markedly, and peripheral edema appeared. Meanwhile, the bilirubin concentration in the blood rose continuously every day, ultimately to a maximum of 20 mg/dL (Fig. 3). Likewise, larger hematomas on the abdomen indicated steadily worsening coagulation. The continued platelet concentrate infusions, AT III, fibrinogen, and vitamin K substitutions were not proving to be effective. On day 23, signs of hepatic encephalopathy (HE) with flapping tremors in both hands appeared for the first time. On the following day, the patient was disoriented, corresponding to a grade 2 HE. Blood ammonia was measured at 114 µg/dL. In addition, rising creatinine levels indicated acute kidney injury, which, after the exclusion of other causes, was to be considered a hepatorenal syndrome. The patient now showed all the cardinal symptoms of acute liver failure: icterus, coagulopathy, encephalopathy, and additionally renal failure. The severity of the encephalopathy varied from day to day, and the patient became increasingly difficult to manage, so she had to be transferred to the intensive care unit on day 28. To confirm the need for therapy with defibrotide, a liver biopsy was to be performed on day 32 to histologically confirm the diagnosis of VOD. A sonographic image of the liver prior to biopsy is shown in Fig. 4. After appropriate substitution of platelets and coagulants, a sonography-guided percutaneous liver biopsy was performed, primarily without complications. Shortly thereafter, a significant drop in hemoglobin levels occurred. The emergency CT angiography showed intra-abdominal hemorrhage from the sight of the liver biopsy (Fig. 5). Several packed red blood cells (RBCs) and fluids were transfused, and the bleeding was stopped by interventional particle embolization. The patient was Hb stable for a short period of time until the bleeding recurred. She subsequently went into hemorrhagic shock with lactic acidosis despite further transfusions and consecutively died.

**Fig. 6:**
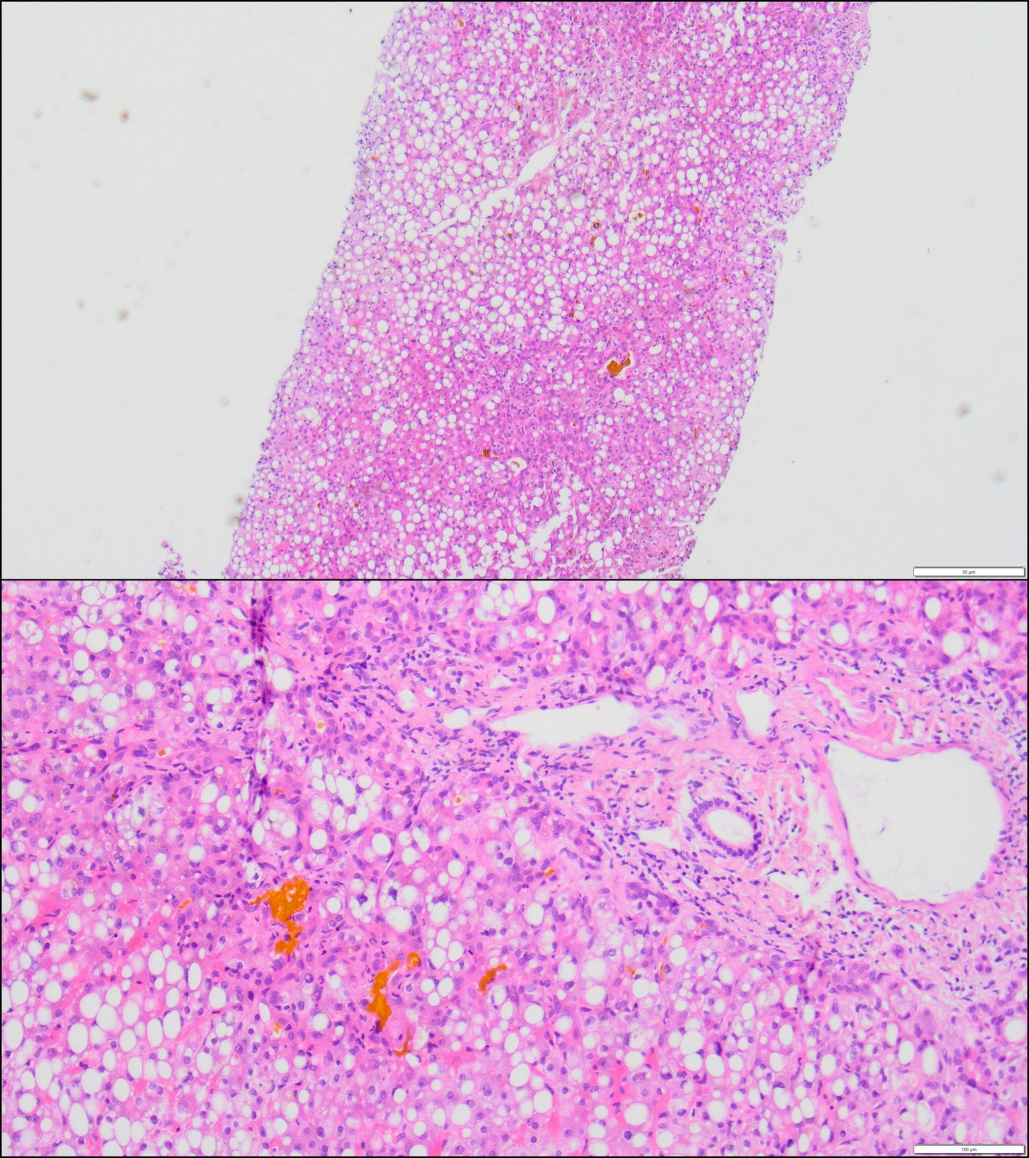
Histological section of liver biopsy (HE stain) showing marked microvesicular and macrovesicular fatty degeneration and marked cholestasis with mild lobular inflammation

The evaluation of the liver biopsy did not show any signs of centrilobular necrosis, which would be the typical picture of VOD. Instead, it showed steatohepatitis as the closest compatible with drug-induced toxic liver damage (Fig. 6). A blood sample taken on day 23 subsequently showed a pegaspargase activity of 134 U/L, which corresponds to prolonged activity.

## Discussion

### Pros and cons of using inotuzumab in relapsed/refractory ALL

The prognosis for relapsed/refractory ALL has been historically very poor. Overall survival at 10 years with chemotherapy was less than 10% in relapsed patients [12]. Relapse after HSCT is associated with an even poorer prognosis [13]. In B-precursor leukemias, treatment with newer immunotherapies such as blinatumomab and inotuzumab ozogamicin (InO) has become more established than standard chemotherapy. Two international randomized trials showed that these two agents achieved significant improvement in CR/CRi rates and median overall survival (OS).

In the randomized phase 3 INO-VATE trial, InO improved the CR/CRi rate from 30.9 to 73.8% and prolonged the median OS from 6.2 to 7.7 months compared to standard of care (SoC) [14]. However, these improvements do not come without a cost. Although the rate of adverse events (AEs) is not markedly increased, the InO arm showed both more dose reductions due to AEs, more temporary interruptions, and more complete discontinuations of study participation due to AEs. In addition, the InO arm showed an increased risk of hepatotoxic AEs (50.6% vs. 36.4%) including a markedly increased risk of VOD (14.0% vs. 2.1%). Hepatotoxicity is especially elevated after follow-up HSCT. In reverse order, the risk is also increased. In a study of sonographic liver stiffness, Marconi examined 16 patients with r/r-ALL before and after treatment with InO [15]. Of these, 6 patients were post-HSCT before receiving InO. Of these, one patient developed severe VOD after InO. Bertamini et al. present a case study of two patients with EM-ALL who showed a very good response (mCR and deep mR) to InO [10]. Furthermore, a case collection showed that InO is effective in patients with r/r B-ALL and extramedullary manifestations [16]. In this analysis, 42% achieved CR after the first cycle of InO and 55% overall after the second cycle; the long-term outcome was however poor. These results provide a rationale to consider therapy with InO in cases of extramedullary relapsed ALL.

### Reasoning for combining chemotherapy and inotuzumab ozogamicin

In our case, we present a patient with a very advanced disease, with 2nd relapse after transplantation, after the failure of DLIs and blinatumomab, and with extramedullary involvement. As the patient was sufficiently physically fit, an intensive therapy concept was decided upon, and the chemotherapeutic backbone was combined with InO in order to increase efficacy. The model for this included a phase II trial from the MD Anderson Cancer Center, which treated patients with r/r ALL with a combination of dose-reduced chemotherapy (mini-hyper-CVD: cyclophosphamide, vincristine, dexamethasone) and InO: Jabbour et al. conducted a phase 2 clinical trial of 96 adults (median age of 37) with r/r ALL [17]. The reported overall response rate was 80% with the 3-year OS rates at 33%. VOD occurred in 10% with a higher rate in those who received HSCT. This study demonstrated that a combination of InO with a dose-reduced chemotherapy is safe and effective in patients with r/r ALL. Interestingly, it was reported that the InO-related risk of hepatic AEs and VOD can be lowered by fractionated dosing of InO. In another ongoing study, the INITIAL-1 trial, InO is investigated as a replacement for conventional induction chemotherapy in patients aged > 55 years with newly diagnosed Ph-negative B-ALL. In a preliminary analysis of the data from 43 patients, the overall response rate was 100% with MRD response up to 90% (MRD negativity or MRD-levels < 10^−4^) [18].

### Causes of liver injury and failure in ALL treatment after HSCT

Patients who undergo allogenic HSCT belong to one of the most complex patient collectives. This is associated with several conditions that carry the risk of liver injury and failure, especially after myeloablative conditioning. The most common causes are drug-induced liver damage, e.g., through chemotherapeutic medications. Alkylating agents in particular are linked to an increased risk of VOD, especially when more than one alkylating agent is used, and in particular when in combination with InO [14]. Further drugs include several antifungals (azoles, echinocandins, and liposomal amphotericin B), calcineurin inhibitors (cyclosporin, tacrolimus), NSAIDs and paracetamol, and antidepressants. Infectious causes in patients (especially immune compromised) may include hepatotropic viruses such as the hepatitis viruses A, B, C, D, and E, Epstein-Barr-Virus (EBV), cytomegalovirus (CMV), varicella-zoster virus (VZV), human herpesvirus 6 (HHV-6), coxsackie virus, adenovirus, mumps, and rubella virus. Bacterial and fungal causes include mycobacterium tuberculosis, salmonella and shigella, several spirochaetales and rickettsiae, candida, and cryptosporidium. Further causes may be autoimmune hepatitis or alloimmune hepatitis (i.e., liver GVHD), hemophagocytic lymphohistiocytosis (HLH), iron overload, and malignant causes, either infiltration through the underlying hematological disease or newly developed primary liver cancer. Other less frequent complications include nodular regenerative hyperplasia and focal nodular hyperplasia [6].

### Toxicity profile and assessment of pegaspargase

Pegaspargase (Oncaspar®) is the PEGylated form of L-asparaginase with prolonged activity. Pegaspargase is an essential part of pediatric-based standard therapy of ALL and administered in combination with other chemotherapeutic drugs in induction and/or consolidation, e.g., methotrexate, daunorubicin, or corticosteroids.

Asparaginase has a complex toxicity profile [19]. One of the main side effects of asparaginase is the provocation of an anaphylactic reaction. By PEGylating asparaginase, this risk is significantly minimized. However, there remain other side effects that need to be considered: Among the most common are skin rash, an increase in transaminases and bilirubin, and complex interactions with the coagulation system as evident from decreases of AT III and fibrinogen a prolongation of the activated partial thromboplastin time. Liver toxicity and fatty liver disease often occur, rarely hepatic necrosis, icterus, cholestasis, and liver failure. Neurological disorders ranging from confusion to seizures are rare. Very rarely, reversible posterior leukoencephalopathy syndrome (RPLS) may occur. Pegaspargase is myelotoxic and can suppress all three cell lineages. It often leads to thrombosis, embolism, and hemorrhage.

### Comparison between percutaneous and transvenous liver biopsies

If a histological confirmation of the diagnosis is necessary, a transjugular liver biopsy (TJLB) should be performed with sufficient platelet count and after optimization of plasmatic coagulation. TJLB is a safe and effective procedure, even in critically ill patients or in patients with severe coagulopathy. The main benefit of the transjugular access is explained by the direction in which the biopsy is performed: from inside the hepatic vein into the liver parenchyma, without traversing the liver capsule. If bleeding does occur, it is directed into the venous system, thereby preventing blood loss. Although puncture and rupture of the liver capsule remain possible, the risk is minimized. In a systematic review of 60 studies published between 1978 and 2006 by Kalambokis et al., the authors found that major complications occurred in 0.5% of biopsies. Intraperitoneal hemorrhage occurred in 0.2% [20]. In one retrospective study with 952 patients, the rate of major complications (including intraperitoneal bleeding) was 1%. Interestingly, there was no statistically significant difference in complication rate between patient groups stratified by platelet count and INR, suggesting that TJLB is safe also in patients with severe coagulopathy [21]. In contrast, a retrospective analysis of 6613 performed percutaneous image-guided liver biopsies showed a major complication rate of 0.7%. The rate of severe hemorrhage requiring transfusion and/or angiographic intervention was 0.5% [22]. Both methods show similar low rates of major complications. However, a selection bias must be pointed out, as patients with contraindications for percutaneous liver puncture (such as coagulopathy, thrombocytopenia, or ascites) may be referred to TJLB.

### Conclusions for sequential inotuzumab, methotrexate, and pegaspargase resulting from this case

This case raises the question of the use of further chemotherapy after InO-based induction therapy, particularly, the use of in combination with chemotherapeutic agents with hepatotoxic effects such as methotrexate and pegaspargase. The published literature on InO in combination with a chemotherapeutic backbone mainly uses mini-hyper-CVD [17]. This regimen contains dose-reduced dexamethasone and cyclophosphamide and substantially dose-reduced MTX (250 mg/m^2^). In comparison, the GMALL regimen for older patients includes high-dose MTX (1000 mg/m^2^; 500 mg/m^2^ if > 70 years) and pegaspargase administration (1000 U/m^2^; 500 U/m^2^ if > 70 years). All substances have a hepatotoxic side effect profile. This regimen was feasible with excellent interim results in de novo ALL [18]. The situation may be different in relapse after HSCT after extensive prior therapy. In these situations, many of the recognized risk factors for VOD will most likely be present.

Our case does not represent a typical treatment according to GMALL regimens because the patient had already received an allogenic stem cell transplantation, developed a relapse, and therefore received an individualized relapse therapy. No systematic data on such individualized approaches are available. We refer however to the GMALL Initial-1 trial, which we considered for the treatment decision for this patient. In the Initial-1 trial for older patients with newly diagnosed ALL, patients received an induction therapy with 3 cycles of inotuzumab followed by standard consolidation cycles including intermediate-dose methotrexate and pegaspargase. Preliminary data reported one case of VOD during Inotuzumab induction [23] and an overall quite good tolerance in de novo ALL. This situation is however not comparable with a situation of relapse after HSCT.

To increase safety, patients with an increased risk of DILI and VOD must be identified at an early stage. This also includes a review of all previous therapies and their liver-toxic potential, even if they were administered years ago: Was there a history of known DILI, under which substances, and in what severity? If a patient is to receive InO, they are at high risk for VOD. Prophylaxis and management of adverse events should be performed as outlined in the EBMT guidelines [6, 24] or in the expert panel review from Kebriaei et al. [11] Liver function must be checked by means of laboratory chemistry tests before starting therapy, and structural signs of liver damage must be ruled out by means of imaging procedures such as sonography. During therapy, liver function parameters, bilirubin, and transaminases should be monitored at close intervals. For certain drugs, especially those with longer half-lives such as MTX or pegaspargase, drug monitoring should be done. If a DILI occurs, all non-invasive examination methods should first be exhausted to differentiate and confirm the diagnosis. The risk of invasive procedures such as liver puncture for histological evaluation must be critically weighed against their benefit. A transjugular hemodynamic assessment can also be performed prior to liver puncture. This allows to reliably measure the hepatic venous pressure gradient (HVPG). An HVPG above 10 mmHg is highly specific (> 90%), with moderate sensitivity (52%) [25]. If VOD can be diagnosed by the EBMT criteria and therapy with defibrotide has been initiated, the additional risk of bleeding must be considered when performing invasive procedures. If a histological confirmation of the diagnosis is essential, the biopsy should be performed transjugular with sufficient platelet count and after optimization of coagulation.

## Summary

In this case, a 58-year-old patient presented with an extramedullary B-ALL relapse after prior allogenic HSCT and blinatumomab therapy. The conceived salvage therapy combined InO-based induction followed by consolidation with high-dose MTX and pegaspargase based on the GMALL protocol for older ALL patients. The treatment resulted in a drug-induced acute liver failure. Although clinical signs indicated an InO-related VOD, a liver biopsy revealed chemotherapy-induced liver damage. The patient died of hemorrhagic shock through postinterventional bleeding after liver biopsy. In conclusion, although InO shows promising results in the therapy of r/r ALL and de novo with and without additional chemotherapy, increased hepatic toxicity may occur in heavily pretreated patients with relapse after HSCT. The risk may be increased by additional hepatotoxic drugs such as MTX and pegaspargase.

## Data Availability

The datasets generated and analyzed in the current study are available from the corresponding author on reasonable request.
